# Allo-immunisation fœto-maternelle sévère: à propos d´un cas et revue de la littérature

**DOI:** 10.11604/pamj.2021.38.67.26353

**Published:** 2021-01-20

**Authors:** Sara Ait Souabni, Belhaddad El Habib, Ihsane Oubahha, Jihane El Baqali, Abderrahim Aboulfalah, Abderraouf Soummani

**Affiliations:** 1 Université Cadi Ayyad, Faculté de Médecine et de Pharmacie de Marrakech, Marrakech, Maroc,; 2 Service de Gynécologie-Obstétrique, CHU Med VI Marrakech, Marrakech, Maroc

**Keywords:** Allo-immunisation fœto-maternelle, hémolyse fœtale, anasarque fœto-placentaire, à propos d´un cas, Maternal alloimmunization, fetal hemolysis, feto-placental anasarca, case report

## Abstract

Avant les années 1970, l´allo-immunisation fœto-maternelle était la principale cause de décès périnatal. Actuellement elle est devenue plus rare, grâce à un dépistage des grossesses à risque et à un meilleur suivi de celles-ci. L´avènement du Doppler intracrânien a constitué un véritable tournant dans le suivi de ces grossesses, vu qu´il représente un moyen diagnostic fiable et non invasif du degré d´anémie fœtale, ce qui permet de prendre la décision de transfusion in-utéro. L´immunoprophylaxie anti-D a également joué un rôle important dans la prévention des anémies hémolytiques fœtales et néonatales, et son administration est actuellement bien codifiée. Une prise en charge adéquate permet d´éviter les conséquences de l´allo-immunisation sur le fœtus et le nouveau-né, ainsi que de diminuer les risques de cette affection sur les grossesses ultérieures. Nous rapportons le cas d´une allo-immunisation fœto-maternelle rhésus grave sur grossesse non suivie compliquée d´anasarque fœto-placentaire.

## Abstract (en)

Prior to 1970, maternal alloimmunization was the leading cause of perinatal death. Currently, it has become rarer thanks to screening and monitoring in high-risk pregnancies. The advent of transcranial doppler has been a turning point in the monitoring of these pregnancies, as it is a reliable, non-invasive method for the diagnosis of fetal anemia. This helps clinicians decide whether or not to perform intrauterine transfusion. Anti-D immunoprophylaxis has also played an important role in preventing fetal and neonatal hemolytic anemia and its administration is currently well codified. Adequate management helps to avoid the effects of alloimmunization on the fetus and newborn as well as to reduce the risks of alloimmunization in subsequent pregnancies. We here report a case of severe fetomaternal rhesus (Rh) alloimmunization during unmonitored pregnancy complicated by fetoplacental anasarca.

## Introduction

L´allo-immunisation fœto-maternelle est une affection devenue de plus en plus rare mais dont les conséquences sont graves et pouvant être létales. Ces répercussions fœtales peuvent néanmoins être prévenues par un bon suivi de grossesse et une instauration d´une immunoprophylaxie adéquate. Un grand progrès a été réalisé dans le domaine du suivi et du traitement des allo-immunisations, aussi bien in-utéro ou dans la période néonatale, si bien que même à des stades avancés, une prise en charge rigoureuse permet d´éviter les états d´anasarque et les morts fœtales in utéro.

## Patient et observation

Il s´agit d´une patiente âgée de 27 ans, de groupage AB rhésus négatif, sans antécédents médico-chirurgicaux notables, 3^e^ geste, 3^e^ pare, 1 seul enfant vivant accouché par voie basse, 1 décès néonatal dans un contexte d´incompatibilité fœto-maternelle rhésus. Elle s´est présentée à 33 semaines d´aménorrhée pour accouchement de sa grossesse. Il s´agissait d´une grossesse non suivie. A l´examen clinique, la parturiente avait une hauteur utérine à 36 cm, et elle était en travail avec une dilatation à deux doigts.

**Figure 1:**
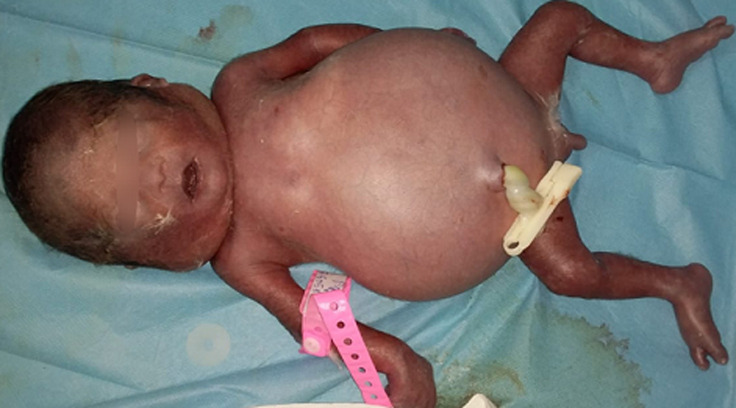
nouveau-né en anasarque

Une échographie obstétricale a été réalisée en urgence, ayant révélé une grossesse monofœtale évolutive en présentation siège avec un diamètre bipariétal (BIP) à 89mm, une longueur du fémur (LF) à 63 et une circonférence abdominale à 4.02 cm, avec polysérite, épaississement placentaire et hydramnios. Le fœtus était bradycarde sur l´échographie obstétricale, ce qui a justifié la réalisation d´une césarienne pour souffrance fœtale sur anasarque fœto-placentaire. Le nouveau-né était à réactif avec un score d´APGAR à 2/10^e^ passé à 4/10^e^ à la 5^e^ minute. Une demande de sang a été faite. L´hémogramme a montré une hémoglobine à 5,8 g/dl. L´exsanguino-transfusion n´a pas pu être réalisée car le nouveau-né est décédé après 45 minutes de vie extra-utérine (Figure 1).

## Discussion

L´allo-immunisation érythrocytaire fœto-maternelle est définie par la présence chez une femme enceinte d´allo-anticorps dirigés contre des antigènes de groupe sanguin présents sur les hématies du fœtus et hérités du père [1]. Il existe deux composantes du système immunitaire, chacune opérant de façon indépendante l´une de l´autre: l´immunité innée: elle est rapide, et représente la première ligne de défense mise en place contre les infections. Elle est cependant non spécifique [2]. L´immunité adaptative: bien qu´elle soit lente, elle est hautement spécifique, capable de reconnaître et d´éliminer sélectivement les molécules étrangères. Elle est caractérisée également par sa mémoire immunitaire [2]. Elle met en jeu plusieurs types de lymphocytes: lymphocytes B, plasmocytes, lymphocytes T8 et T4, ainsi que les cellules présentatrices d´antigène. Elle est capable d´induire une réaction immunitaire soit à médiation cellulaire, soit à médiation humorale (impliquant les anticorps).

Tout antigène érythrocytaire est défini par rapport à sa reconnaissance par un anticorps polyclonal spécifique d´origine humaine. Les anticorps irréguliers mis en évidence sont majoritairement dirigés contre les antigènes Rh (surtout anti-RH1, puis anti-RH4, anti-RH3), plus rarement contre les antigènes du système KEL (anti-KEL1) ou d´autres systèmes de groupe sanguin érythrocytaires (JK, FY, MNS) [1]. Le pouvoir immunogène des antigènes érythrocytaires varie d´un système de groupe sanguin à un autre, et d´un antigène à un autre au sein d´un même système de groupe sanguin. L´antigène RH1 (D) est le plus immunogène des antigènes érythrocytaires [2, 3].

Lorsqu´il existe un passage transplacentaire d´hématies fœtales RhD positif dans la circulation maternelle d´une femme RhD négatif, le système immunitaire synthétise des anticorps IgG anti-D. Les anticorps maternels traversant le placenta vers la circulation fœtale provoquant une hémolyse et une anémie chez le fœtus RhD positif [4]. En effet, il existe un mécanisme actif lié à l´interaction entre le site Fc des anticorps RH1 et les récepteurs Fc présents au niveau du placenta, les anticorps anti-RH1 vont venir se fixer sur les antigènes RH1 présents à la surface des hématies fœtales. Les complexes immuns ainsi formés vont venir activer les macrophages fœtaux en s´amarrant à leur récepteur Fc. Une destruction des hématies fœtales s´ensuit alors par phagocytose ou lyse de contact au niveau de la rate et de foie fœtal.

### Suivi des grossesses à risque

Le dépistage débute par la détermination du groupage maternel [5]. Chez les femmes de rhésus négatif, il est recommandé de vérifier systématiquement le groupage du conjoint. Si le conjoint est de RhD négatif, il n´y a pas besoin d´immunoprophylaxie [6]. La recherche des agglutinines irrégulières (RAI) est la technique de choix pour la surveillance des grossesses à risque. En effet, l'absence d'agglutinine irrégulière élimine toute atteinte fœtale [7]. Toutes les femmes enceintes doivent bénéficier d'une recherche des agglutinines irrégulière en début de grossesse. Pour les femmes de groupe RhD négatif, cet examen doit être répété chaque mois. La positivisation des RAI au cours de la grossesse témoigne de la survenue d'allo-immunisation dont il faut apprécier la gravité. La titration des agglutinines irrégulières est réalisée dès que la recherche des agglutinines irrégulières est positive [8]. Au-delà d'un titre de 8, il est préférable de réaliser un dosage pondéral des anticorps [9]. Le dosage pondéral permet d´exprimer la concentration en μg/ml des IgG anti-RH. Il permet une approche de la concentration réelle en IgG anti-RH dans le sérum maternel. Le seuil dangereux est de 1μg/ml [10].

### Prévention de l´allo-immunisation

La prévention ciblée, instaurée depuis 1970, consiste à administrer l´immunoglobuline anti-D dans les 72 heures en cas d´évènement potentiellement immunisant. Au-delà, un bénéfice peut être espéré jusqu´à 30 jours [8]. Il s´agit, au premier trimestre des fausses couches, métrorragies, GEU, IVG, traumatisme abdominal, biopsie de villosités choriales, et aux trimestres suivants en cas d´amniocentèse, ponction de sang fœtal, fausse couche spontanée, métrorragies, cerclage du col, version par manœuvres externes, mort fœtale in utero (MFIU), traumatisme abdominal, IMG, et aux mères RhD négatif de nouveau-nés RhD positif. [6] La dose minimale d´anti-D administrée est de 250 UI au premier trimestre, et de 500 UI à partir de la 20^e^ semaine [7]. Le test de Kleihauer est un test cytochimique permettant la recherche et la quantification d´hématies fœtales dans la circulation sanguine maternelle. Son principe repose sur la résistance de l´hémoglobine fœtale en milieu acide, contrairement à l´hémoglobine adulte qui est soluble dans ce milieu. Ce test est réalisé à partir d´un échantillon de sang maternel prélevé sur un tube avec anticoagulant à partir de 10-11 semaines de grossesse [11]. Le test de Kleihauer n´est pas nécessaire avant l´injection d´immunoglobulines au premier trimestre [8]. Au cours du deuxième trimestre, et dans des circonstances pouvant entraîner un passage important d´hématies fœtales, la posologie des immunoglobulines est guidée par ce test (Kleihauer).

Au cours du 3^e^ trimestre, chez toute femme Rh D négatif, non immunisée contre l´antigène D et dont le fœtus est connu ou présumé Rh D positif, doit se voir proposer une injection d´immunoglobulines anti-D de 300 µg par voie intramusculaire à 28 SA (± 1 semaine) [8]. Après l´accouchement, la prophylaxie RH doit être appliquée quand l´enfant est de Rh+ alors que la mère est de Rh- non immunisée. Ceci nécessite au préalable la double détermination des groupes sanguins ABO-RH1 du nouveau-né, la RAI maternelle à l´accouchement et le test de Kleihauer sur le sang maternel prélevé au moins une heure après la délivrance [10].

**Conséquences fœtales et néonatales de l´allo-immunisation:** l´hémolyse a deux principales conséquences: l´anémie hémolytique, touchant aussi bien le fœtus que le nouveau-né; l´hyperbilirubinémie, avec risque d´ictère nucléaire, concernant le nouveau-né.

**L´anémie:** jusqu´au milieu du deuxième trimestre, le fœtus a des besoins modérés en oxygène, et donc une bonne tolérance à l´anémie [11]. Il met en place plusieurs mécanismes compensatoires: hyperhématopoïèse, grâce à la stimulation de la sécrétion d´érythropoïétine, d´origine extra-médullaire ce qui a pour conséquence une hépatosplénomégalie; augmentation du débit sanguin par tachycardie fœtale et mise en place d´une circulation d´épargne avec redistribution préférentielle vers les organes nobles que sont le cœur et le cerveau puisque l´anémie entraîne une hypoxie tissulaire [11].

Au-dessous d´une valeur proche de 7 g/dl au cours du 2^e^ trimestre et de 9 g/dl au 3^e^ trimestre, l´accroissement du débit cardiaque (objectivable par vélocimétrie) s´accompagne d´anomalies du rythme cardiaque fœtal (RCF) puis d´épanchements liquidiens réalisant une anasarque fœto-placentaire [12]. Cette dernière est définie par une infiltration liquidienne anormale concernant au minimum deux séreuses (plèvre, péritoine, péricarde) associée à un œdème cutané généralisé [11]. Ce tableau est d´abord réversible par transfusion (stade d´insuffisance cardiaque fonctionnelle), puis difficilement réversible après que l´état d´anoxie chronique a induit des lésions et des remaniements cellulaires profonds (stade d´anasarque lésionnel) [12]. La résistance du fœtus à l´anémie semble plus importante au cours du début du 2^e^ trimestre, durant lequel l´anasarque s´exprime chez des fœtus ayant des taux d´hémoglobine compris entre 2 g/dl et 6 g/dl. L´anémie peut se constituer très rapidement au cours des 8^e^ et 9^e^ mois et les signes d´anasarque peuvent être frustes avant la survenue du décès in utero [12]. Le pronostic d´un fœtus en anasarque est sévère avec une évolution vers la Mort Fœtale In Utero (MFIU) et une mortalité périnatale importante [11].

### L´ictère nucléaire

A la naissance, l´hémolyse se poursuit pendant trois mois, ce qui correspond à la durée de vie des IgG maternelles transmises au fœtus. Pour être éliminée, l´hème de l´hémoglobine est transformé en biliverdine, bilirubine libre, puis en bilirubine conjuguée. La transformation de la bilirubine libre en bilirubine conjuguée se fait au niveau hépatique, afin d´être éliminée par les selles et les urines. Durant la grossesse, ce processus de conjugaison est fait par l´intermédiaire du trophoblaste [12]. Après l´accouchement, il existe une immaturité hépatique fœtale ce qui le rend incapable de se débarrasser de l´excès de bilirubine induit par l´hyperhémolyse. La bilirubine libre s´accumule donc dans la circulation du nouveau-né, et peut induire un ictère nucléaire, qui est la conséquence de l´action toxique de la bilirubine non conjuguée sur les neurones des noyaux thalamiques, sous-thalamiques, du tronc cérébral et du cervelet [12].

### Surveillance fœtale

La surveillance porte essentiellement sur les éléments suivants: le titrage et le dosage pondéral des anticorps ainsi que leur cinétique. Le taux des anticorps maternels est assez bien corrélé au risque hémolytique. Les valeurs de titre et de dosage pondéral sont alors à interpréter en fonction du terme de grossesse [8]; l´échographie à la recherche de signes évoquant une anasarque débutante, témoignant d´une insuffisance cardiaque fœtale liée à l´anémie. Le syndrome précoce de décompensation est caractérisé par la découverte d´un ou plusieurs des éléments suivants: anses intestinales anormalement échogènes, visualisation de la paroi intestinale, lame d´ascite, hépatomégalie, image en double contour cutané discret au niveau du crâne, épanchement péricardique, excès de liquide amniotique, augmentation de l´épaisseur du placenta et augmentation du diamètre de la veine ombilicale dans son trajet intra- ou extrahépatique [8]; La mesure par Doppler du pic systolique du flux sanguin de l´artère cérébrale moyenne représente actuellement le paramètre de choix dans l´évaluation du degré d´anémie chez le fœtus entre 16 et 35 semaines d´aménorrhée [1]. Le chiffre obtenu est rapporté à la valeur médiane de la population du même âge gestationnel (courbe de Mari) pour établir le Multiple of Median (MoM) du fœtus qui est bien corrélé au taux d´hémoglobine fœtale [1, 13]. Pour les grossesses qui ont atteint 16-24 semaines, ou lorsqu'un titre d'anticorps critique est atteint, l'anémie fœtale est surveillée à l'aide du Doppler du pic systolique du flux sanguin de l´artère cérébrale moyenne toutes les 2 semaines pour la stratification du risque [13]. Une valeur de plus de 1,5 MoM est très sensible pour l´évaluation du risque d´anémie fœtale, avec un taux de faux positifs de 12% [13].

### Prise en charge in utéro en cas d´anémie hémolytique

Elle repose essentiellement sur la transfusion in utero. La voie intrapéritonéale a été introduite à partir de 1963 par Liley avant l´avènement de l´échographie [14]. Elle se faisait sous repérage radiologique du fœtus avec injection de produit de contraste.

La transfusion par voie intravasculaire a été proposée en 1981 et 1982 par Rodeck *et al*. et par Bang, et elle a permis une meilleure prise en charge de l´anémie hémolytique fœtale, ainsi qu´une diminution de sa morbi-mortalité [15]. L´abord vasculaire doit préférentiellement se faire au niveau de la portion intra-abdominale de la veine ombilicale ou au niveau de l´insertion placentaire du cordon afin de diminuer les risques de complications [15].

Auparavant, la décision de transfusion in utero se prenait après réalisation d´une amniocentèse avec mesure de la concentration en bilirubine dans le liquide amniotique. Néanmoins, il s´agissait d´une technique invasive, nécessitant souvent d´être répétée. En 2000, Mari *et al*. [16] ont introduit la mesure du pic systolique de vélocité de l´artère cérébrale moyenne fœtale qui est devenue le gold standard dans l´indication de la transfusion in utéro [15].

La première transfusion intravasculaire peut être réalisée à partir de 18-20 SA au plus tôt. Le rythme des transfusions dépend du taux d´hémoglobine initial et du taux d´hémoglobine atteint à la fin de la transfusion. La ponction de sang fœtal (PSF) permet non seulement d´analyser le sang fœtal, mais aussi de réaliser des transfusions ou exsanguino-transfusions in utéro lorsqu´il y a une indication [8].

La perte moyenne quotidienne d´hémoglobine est aux alentours de 0,3 g. La fréquence des transfusions est à adapter en fonction du suivi de l´évolution du taux d´hémoglobine de l´enfant et du Doppler au niveau de l´artère cérébrale moyenne. En moyenne, ces transfusions itératives sont réalisées toutes les 2 à 4 semaines jusqu´à ce que l´on décide de faire naître l´enfant [8]. La transfusion in utero est considérée comme étant une procédure sûre dont les complications restent rares et dont les bénéfices sont majeurs [15].

### Décision d´extraction

L´accouchement prématuré est réalisé avant 34 semaines en cas de souffrance fœtale ou d´échec de la transfusion fœtale. L´idéal étant d´atteindre au moins 30 SA pour réduire les risques de mortalité et de morbidité postnatales. Il est nécessaire, si le principe d´une extraction fœtale prématurée est envisagé de réaliser une corticothérapie de maturation pulmonaire. Après 34 SA, une naissance est facilement envisagée si le risque fœtal est élevé [8].

### Prise en charge néonatale

La prise en charge post-natale doit se faire en unité de soins intensifs néonataux. Elle doit être précoce et ciblée. Elle s´articule autour de deux objectifs principaux : La correction de l´anémie et la prévention de l´ictère nucléaire. Les thérapeutiques envisageables sont la transfusion sanguine en cas d´anémie mal tolérée, la photothérapie et l´exsanguino-transfusion. Cette dernière est réservée aux tableaux d´anasarque fœto-placentaire avec hyperbilirubinémie importante et risque accru d´ictère nucléaire, ou en cas d´hyperbilirubinémie résistant à la photothérapie [17].

## Conclusion

Un suivi adéquat des grossesses à risque d´allo-immunisation rhésus, ainsi qu´une prise en charge néonatale appropriée sont les meilleurs garants pour éviter les conséquences délétères et souvent létales de cette affection. Un grand progrès a été réalisé dans ce domaine et qui a permis une amélioration significative du devenir de ces grossesses autrefois vouées à la mort fœtale in utéro ou au décès néonatal.
